# P-1169. Disseminated Pediatric Coccidioidomycosis at an Academic Center in Southern Arizona (2013 - 2023)

**DOI:** 10.1093/ofid/ofae631.1355

**Published:** 2025-01-29

**Authors:** Marian Lacy, Jordan Gotwalt, Neil M Ampel, Kareem W Shehab

**Affiliations:** University of Arizona College of Medicine, Tucson, Arizona; University of Arizona College of Medicine - Tucson, Tucson, Arizona; University of Arizona, Tucson, Arizona; University of Arizona, Tucson, Arizona

## Abstract

**Background:**

There have been few reviews of pediatric disseminated coccidioidomycosis, particularly regarding presentation. A better understanding of this may enable more timely diagnosis and improve management in children.

**Methods:**

We identified 157 children with coccidioidomycosis seen in the pediatric infectious diseases practice at University of Arizona – Banner University Medical Center in Tucson, AZ, between 2013 – 2023. Criteria included: age under 18, seen by a pediatric infectious diseases specialist, with a diagnosis of coccidioidomycosis in the consultation note. This study includes the subset of children with a diagnosis of disseminated coccidioidomycosis. Cases were excluded if the electronic medical record did not document at least one of the following supporting diagnostics: positive coccidioidal IgM or IgG by EIA, culture positive for *Coccidioides*, or histopathological findings characteristic of coccidioidomycosis. Demographic, laboratory, and radiologic data as well as clinical characteristics were collected from review of the electronic medical record.

**Results:**

Among 157 patients, 28 (17.8%) had disseminated disease. Median age at diagnosis was 11.7 years (range 7 months to 17 years of age). 64% were male. Musculoskeletal disease was the most common extrapulmonary organ system involved (15 patients), followed by skin (11), central nervous system (4), abdominal organs and viscera (2), pericardium (1) and bloodstream (1). Sixteen (57%) had no comorbidities. The chest radiograph was normal in 10 of 26 (38%) where it was performed. Seventeen (61%) had a negative coccidioidal EIA IgG serology, and 3 patients (11%) had negative IgM and IgG serologies on presentation. Only 3 (11%) had an elevated absolute eosinophil count (above 600/µL).

**Conclusion:**

Disseminated coccidioidomycosis affects children of all ages and can involve many different organ systems, particularly musculoskeletal, skin, and central nervous system. It should be considered in the appropriate clinical setting even in patients with negative coccidioidal serologies, normal chest x-rays, normal eosinophil counts, or no underlying illness.

**Disclosures:**

**All Authors**: No reported disclosures

